# New Insight into Neuropathic Pain: The Relationship between α7nAChR, Ferroptosis, and Neuroinflammation

**DOI:** 10.3390/ijms25126716

**Published:** 2024-06-18

**Authors:** Fangting Luo, Cheng Huang

**Affiliations:** 1 School of Public Health and Health Management, Gannan Medical University, Ganzhou 341000, China; luofangting@gmu.cn; 2 Department of Physiology, School of Basic Medicine, Gannan Medical University, Ganzhou 341000, China; 3 Pain Medicine Research Institute, Gannan Medical University, Ganzhou 341000, China

**Keywords:** neuropathic pain, neuroinflammation, α7nAChR, ferroptosis, microglia

## Abstract

Neuropathic pain, which refers to pain caused by a lesion or disease of the somatosensory system, represents a wide variety of peripheral or central disorders. Treating neuropathic pain is quite demanding, primarily because of its intricate underlying etiological mechanisms. The central nervous system relies on microglia to maintain balance, as they are associated with serving primary immune responses in the brain next to cell communication. Ferroptosis, driven by phospholipid peroxidation and regulated by iron, is a vital mechanism of cell death regulation. Neuroinflammation can be triggered by ferroptosis in microglia, which contributes to the release of inflammatory cytokines. Conversely, neuroinflammation can induce iron accumulation in microglia, resulting in microglial ferroptosis. Accumulating evidence suggests that neuroinflammation, characterized by glial cell activation and the release of inflammatory substances, significantly exacerbates the development of neuropathic pain. By inhibiting microglial ferroptosis, it may be possible to prevent neuroinflammation and subsequently alleviate neuropathic pain. The activation of the homopentameric α7 subtype of the neuronal nicotinic acetylcholine receptor (α7nAChR) has the potential to suppress microglial activation, transitioning M1 microglia to an M2 phenotype, facilitating the release of anti-inflammatory factors, and ultimately reducing neuropathic pain. Recent years have witnessed a growing recognition of the regulatory role of α7nAChR in ferroptosis, which could be a potential target for treating neuropathic pain. This review summarizes the mechanisms related to α7nAChR and the progress of ferroptosis in neuropathic pain according to recent research. Such an exploration will help to elucidate the relationship between α7nAChR, ferroptosis, and neuroinflammation and provide new insights into neuropathic pain management.

## 1. Introduction

Pain is regarded as a hardwired signal of physical disturbance that urges the individual to act and restore the body’s integrity rather than just a sensory and emotional experience [1]. Obstacles and barriers to treatment exist for pain in different groups of patients with different characteristics [2]. Neuropathic pain is characterized by pain resulting from a lesion or disease, which could have an impact on the somatosensory system, as defined by the International Association for the Study of Pain (IASP) [3]. Chronic neuropathic pain can usually be spontaneous and cause an increased response to painful stimuli (hyperalgesia) or a painful response to painless stimuli (allodynia) [4]. According to the IASP, neuropathic pain affects between 6.9% and 10% of the global population, inducing a significant decline in both health and quality of life for patients and placing a heavy strain on families and social healthcare resources [5]. In recent decades, despite notable advancements in the study of neuropathic pain, it remains in its nascent stages, necessitating further comprehensive investigation to fully comprehend its pathogenesis. Due to the limited comprehension of the pathogenesis of neuropathic pain, the clinical application of pharmacological or non-pharmacological interventions does not produce satisfactory outcomes for the majority of patients [6].

An increasing body of research has demonstrated the significant involvement of neuroinflammation in neuropathic pain [7,8,9], with the microglia α7nAChR being a key player in regulating this process [10,11,12]. Ferroptosis is a recently discovered form of cell death controlled by iron [13]. Prior research has indicated a strong connection between ferroptosis and neuroinflammation [14,15]. Studies have shown that neuroinflammation can trigger intracellular iron buildup, speed up iron overload, facilitate the synthesis of reactive oxygen species (ROS), and further result in ferroptosis [16,17], whereas ferroptosis enhances neuroinflammatory reactions [18,19].

Hence, the aim of this article is to explore the interaction among neuroinflammation, microglial α7nAChR activation, and ferroptosis in cases of neuropathic pain to investigate novel therapeutic interventions. To the best of our knowledge, there has been no systematic review that has summarized the association between α7nAChR, ferroptosis, and neuropathic pain. To better understand the potential mechanisms involved in the role of α7nAChR on ferroptosis in neuropathic pain, we elucidate the important role of neuroinflammation, the biochemical and functional features of α7nAChR, and the molecular and metabolic mechanisms of ferroptosis according to recent research.

## 2. Neuroinflammation

Neuroinflammation is characterized by an inflammatory response occurring within the central nervous system (CNS) as a result of both exogenous and endogenous factors [20]. It is an inflammatory cascade reaction involving various immune cells and/or molecules [21]. A neuroinflammatory response is defined by the activation of microglia in the CNS, the infiltration of inflammatory cells, and elevation in secreted pro-inflammatory factors and chemokines, resulting in an inflammatory reaction [22,23].

### 2.1. Role of Microglia in Neuroinflammation

In the CNS, microglia are an essential type of glial cell widely distributed throughout the brain and spinal cord, comprise approximately 5% to 20% of all glial cells, and are recognized as macrophages [24]. Microglia are crucial in initiating and progressing neuroinflammation as the primary component of innate immunity in the CNS [25]. Even in a resting state, the processes of microglia are highly dynamic and perpetually surveil the CNS. Microglia are vital participants in CNS homeostasis, and dysregulation of these sentinels can give rise to neurological disease [26,27].

When there are alterations in the micro-environment and disruptions in homeostasis, both in living organisms and in laboratory settings, particularly during an inflammatory reaction, macrophages have the ability to transform into various functional types [28]. Any factors that affect the homeostasis of the CNS, such as drugs, injury, and pain, can activate microglia [29]. Within the immediate surroundings, microglia have the ability to change their states in reaction to different signals, leading to the activation of either the traditional M1 phenotype (pro-inflammatory type) or the alternative M2 phenotype (anti-inflammatory type). These two types exert opposing effects on promoting and inhibiting inflammatory responses, respectively. After nerve damage causes neuropathic pain, microglial cells change from being inactive to becoming active, especially the M1 phenotype, which releases pro-inflammatory substances like tumor necrosis factor-α (TNF-α), interleukin-1β (IL-1β), and interleukin-6 (IL-6) [30,31]. The release of pro-inflammatory cytokines sensitizes and stimulates nociceptors, which in turn results in local homeostatic changes [32]. In contrast, the M2 phenotype aids in the tissue reconstruction and functional improvement of the nervous system via the secretion of anti-inflammatory substances, including interleukin-10 (IL-10) and transforming growth factor-β (TGF-β) [33].

Another study has suggested that resveratrol promotes the transformation to M2 microglia by reducing activated M1 microglia and ultimately reduces neuroinflammation, indicating that different polarization phenotypes of microglia can determine the outcome of neuroinflammation and affect prognosis [34]. Polysaccharides from Schisandra Chinensis Fructus (SCP2-1) effectively decreased the lipopolysaccharide (LPS)-induced M1 polarization of microglia and encouraged the shift to the M2 phenotype by blocking the nuclear factor-κB (NF-κB) and JNK pathways, finally reducing neuroinflammation [35].

The evidence mentioned above indicates a strong connection between microglia and neuroinflammation. Consequently, modulating the polarization phenotype of microglia serves as a means to impede the inflammatory response, thus exerting a protective influence on the nervous system [36,37,38]. Overall, microglia serve as a double-edged sword in neuroinflammation after neuropathic pain. On the one hand, M2 microglia have a protective effect on the nervous system by releasing anti-inflammatory cytokines. On the other hand, over-activated M1 microglia can disturb the homeostasis of the brain micro-environment, further exacerbating neuroinflammation development.

### 2.2. Neuroinflammation and Neuropathic Pain

A persistent inflammatory response, marked by microglial activation and the release of inflammatory factors, may have a substantial impact on the progression of neuropathic pain. The alterations in inflammatory factors are regarded as a prominent indicator of neuroinflammation [39]. A previous review from 2016 already stated that numerous studies have demonstrated the crucial role of pro-inflammatory cytokines in the regulation of neuropathic pain following peripheral nerve injury [40]. Research indicates that the damage to peripheral nerves can increase the levels of TNF-α, IL-1β, and IL-6 in the CNS [41]. Administering IL-1β via intrathecal injection can cause pain-like behaviors (mechanical allodynia) in healthy rats, but suppressing the IL-1β gene alleviates hyperalgesia in rats with chronic constriction injury (CCI) [42,43]. During the central sensitization process of neuropathic pain, various pro-inflammatory cytokines, such as TNF-α, which arise from M1 microglia, began to increase at the initial stage of the inflammatory response [25]. The nerve growth factor (NGF), bradykinin, prostaglandin (PG), and various other natural pain-inducing agents work in conjunction with the CNS, enhancing nerve cell activity and sensitivity in the dorsal root ganglion (DRG). Meanwhile, there is a delay in the production of anti-inflammatory substances such as IL-10, leading to a decrease in their levels [44,45].

The prevalence of neuropathic pain is substantial, with a wide range of causes and complex underlying mechanisms. Despite this, there remains a dearth of targeted pharmaceutical interventions in clinical settings [40]. Previous research has predominantly focused on elucidating the pathological mechanism of neuropathic pain through the lens of neuronal synaptic remodeling [46]. Research has shown that neuropathic pain arises from peripheral sensitization via the activation of neuronal G protein-coupled receptors, ion channels, and cytokine release. Additionally, it is connected to central sensitization influenced by inflammatory substances and cytokines, primarily exhibited as the stimulation of N-methyl-D-aspartic acid (NMDA) receptors, elevated excitatory synaptic communication, reduced inhibitory synaptic communication, and a compromised performance of the descending regulatory system [47]. In recent years, studies have revealed a significant relationship between microglia and the central sensitization of neuropathic pain [48,49]. Microglia have complicated and ever-changing interactions with neurons, other glial cells, and lymphocytes, which are essential for the onset, progression, and persistence of neuropathic pain [24]. Furthermore, the activation of microglia and their diverse polarization phenotypes, influenced by numerous stimulating factors, contribute to the intricate pathogenesis of neuropathic pain [26]. Therefore, blocking or inhibiting microglial inflammatory responses during subsequent neuropathic pain treatment are critical strategies for intervention.

## 3. α7nAChR

### 3.1. The Biochemical Features of α7nAChR

nAChR is a commonly found receptor belonging to the cysteine-loop receptor superfamily present in the nervous systems. Additional members of this receptor family are the γ-aminobutyric acid type A receptor (GABAAR), glycine receptor (GlyR), and 5-hydroxytryptamine 3 receptor (5-HT3R) [50,51]. Five separate subunits combine to create each of these receptors, forming pentameric ligand-gated ion channels. The human genome contains 16 genes that encode different subunits, such as α1–α7, α9, α10, β1–β4, δ, ε, and γ [52,53,54]. Nicotinic acetylcholine receptors are mainly divided into three subtypes: muscle-type receptors, neuron-type receptors, and non-neuron non-muscle-type receptors. Muscle-type receptors consist of α1, β1, δ, ε, and γ subunits, whereas neuron-type receptors are made up of α2–α7, α9, α10, and β2–β4, among others [55,56].

Compared with other subtypes of nAChRs, α7nAChR exhibits unique functional properties [57], including fast activation and desensitization by agonists (on a millisecond scale), and high calcium permeability [58,59], that differentiates them from most other heterosubunit acetylcholine receptors. When α7nAchR is stimulated in the presynaptic membrane, it can promote the fusion of vesicles and presynaptic membrane, exocytosis, and Ca^2+^ influx, thereby activating voltage-dependent calcium channels to trigger presynaptic membrane depolarization and ultimately promote or stimulate the release of corresponding neurotransmitters [60]. The stimulation of α7nAChR triggers an upregulation in intracellular calcium concentration and then activates the calcium-dependent signaling pathway, thereby increasing the release of glutamate and improving the plasticity of glutamatergic synapses [61]. The homopentameric α7nAChR subtype of neuronal nicotinic acetylcholine receptors is highly prevalent, particularly in microglia. The activation of microglial α7nAChR is shown to have a substantial impact on diminishing the secretion of pro-inflammatory cytokines [62].

### 3.2. Target α7nAChR in the Treatment of Neuropathic Pain

The cholinergic anti-inflammatory pathway, known as CAP, is a newly identified signaling pathway controlled by the vagus nerve and works to suppress the inflammatory response by facilitating communication between nerves and the immune system [63,64]. As the primary component of CAP, α7nAChR has the ability to exert immunoregulatory effects by suppressing the synthesis and release of inflammatory cytokines. Studies have shown that natural acetylcholine (ACh) attaches to α7nAChR on microglia, triggering the JAK2/STAT3 signaling pathway, blocking NF-κB signal transduction, and decreasing the production of inflammatory cytokines like TNF-α and IL-1β, resulting in anti-inflammatory effects [65,66]. During the inflammation caused by LPS, activated α7nAChR can inhibit the M1 polarization of microglia and promote its transformation to the M2 type, indicating that α7nAChR is crucial in controlling the polarization of microglia [67]. Studies on rats with chronic pain induced by CCI or spared nerve injury (SNI) have indicated that the levels of α7nAChR in the spinal cord or DRG are decreased, and activating microglial α7nAChR can lower pro-inflammatory cytokines while upregulating anti-inflammatory counterparts, resulting in a reduction in inflammatory and chronic pain [68]. The activation of α7nAChR by 2 Hz of electroacupuncture (EA) stimulation is found to suppress spinal microglial activation and reduce the expression of IL-1β, ultimately relieving neuropathic pain induced by SNI [69].

The previous evidence indicates that α7nAChR has the potential to facilitate neuroprotection and impede the generation and progression of neuroinflammation via the regulation of microglial activation. Targeting α7nAChR may serve as an effective therapeutic strategy in preventing and managing inflammatory responses, further attenuating neuropathic pain.

## 4. Ferroptosis

Iron is an indispensable trace element for the human body, serving as a vital material foundation for metabolic processes and playing a pivotal role in material conversion, energy provision, growth, and development [70]. Initially, Dixon et al. identified a novel type of cellular demise and formally designated it ferroptosis, a form of cell death that relies on iron [13]. This process involves three primary metabolites, including thiols, lipids, and iron, which can cause the production of iron-dependent lipid peroxides and eventually induce cell death [71]. As a distinct mode of cellular demise, Ferroptosis can be discerned from alternative forms of cell death by its distinctive features, including the buildup of lethal ROS and lipid peroxidation products resulting from iron-dependent processes [72]. Moreover, the pathway leading to ferroptotic cell death is associated with diverse morphological and biochemical attributes [73,74,75,76,77]. Ferroptosis has been demonstrated to be closely associated with the pathophysiological process of numerous neurological disorders, such as neuropathic pain. The potential mechanisms that may be involved include fluctuations in intracellular iron ion concentrations, the modulation of glutamate excitability, and lipid peroxidation. Hence, it is imperative to obtain a thorough comprehension of the impact of ferroptosis on neuropathic pain in order to devise more efficacious therapeutic approaches.

### 4.1. Mechanisms of Ferroptosis

#### 4.1.1. Iron Dyshomeostasis Contributes to Ferroptosis

**Figure 1:**
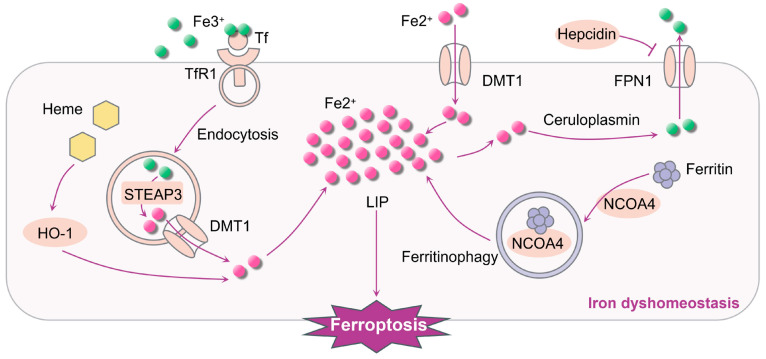
Iron dyshomeostasis implicated in ferroptosis. Fe^2+^ is released through Tf degradation, ferritinophagy, or heme degradation. DMTl mediates the transport of extracellular free Fe^2+^ into the cell. The rise in unbound Fe^2+^ within the cell creates a pool of easily accessible iron. The cell releases Fe^3+^ to the exterior via FPNl. Furthermore, the breakdown of heme and the process of ferritinophagy mediated by nuclear receptor coactivator 4 (NCOA4) can raise the LIP, making cells more susceptible to ferroptosis via the Fenton reaction.

Iron absorption, use, retention, and excretion are intricately linked to the buildup of iron within cells and the constant regulation of iron levels [78]. In the local microenvironment of the body, iron mainly exists in the form of Fe^2+^ and Fe^3+^. Under normal physiological states, the concentration of intracellular iron remains relatively stable. Once iron builds up, it disrupts the balance of iron levels within cells (Figure 1); when iron is overloaded, excessive Fe^2+^ can react with ROOH or hydrogen peroxide (H_2_O_2_), respectively, to produce soluble lipid alkoxy (RO-) or hydroxyl (HO-) radicals, the primary source of ROS produced by the Fenton reaction, and then may further promote the formation of lipid peroxides by obtaining electrons from other molecules, which ultimately leads to ferroptosis [79]. Moreover, proteins associated with iron metabolism, including divalent metal transporter 1 (DMT1), ferroportin-1 (FPN1), transferrin (Tf), transferrin receptor 1 (TfR1), and the iron storage protein ferritin, play major roles in triggering ferroptosis. The typical route for the absorption of transferrin-bound iron (TBI) includes the internalization of iron-rich Tf by TfR1 in order to form endosomes [80,81]. STEAP3 (Six-Transmembrane Epithelial Antigen of Prostate 3) is specifically located within these endosomes. Within the acidic conditions of the endosomes, STEAP3 aids in converting Fe^3+^ to Fe^2+^ [82,83]. Following this, Fe^2+^ is moved from the outer layer of the endoplasmic reticulum through DMT1 into the cytoplasm, which helps create a labile intracellular iron pool (LIP) [84]. Meanwhile, intracellular free Fe^2+^ is converted to Fe^3+^ by ceruloplasmin and then discharged from the cells inside by FPN1. Iron entering microglia is usually stored in ferritin or an unstable iron pool. Under normal conditions, LIP can maintain the dynamic balance of iron [85], and excessive Fe^2+^ can promote ferroptosis. Therefore, numerous proteins involved in regulating cellular iron homeostasis (such as DMT1, FPN1, and TfR1) can affect the sensitivity of the cell to ferroptosis.

#### 4.1.2. System Xc-/GSH/GPX4 Pathway Regulates Ferroptosis

**Figure 2:**
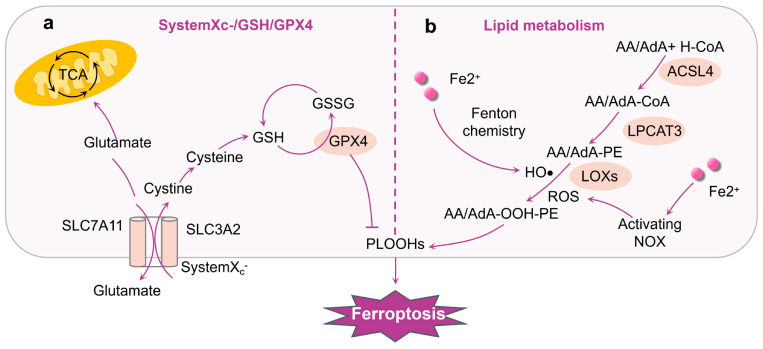
Glutamine metabolism and lipid peroxidation implicated in ferroptosis. (**a**) The primary pathway that inhibits ferroptosis includes the absorption of cystine via the cystine–glutamate antiporter (System X_c_^−^), leading to the production of GSH. GPX4 utilizes GSH as a cofactor to convert phospholipid hydroperoxides into their respective alcohols. GPX4 catalyzes the reduction in PLOOHs by GSH, inducing the inhibition of cell ferroptosis. Mitochondria host a wide range of critical metabolic processes (such as the tricarboxylic acid (TCA) cycle) and are a significant source of ROS. (**b**) The presence of Fe^2+^ in the cytoplasm results in a notable rise in the generation of HO· and ROS, with the transformation of AA/AdA to AA/AdA-PE occurring through a sequential process catalyzed by ACSL4 and LPCAT3. HO·, ROS, or LOXs can enhance the conversion of AA/AdA-OOH-PE into PLOOHs. The cell membrane undergoes phospholipid peroxidation caused by PLOOHs, resulting in cell ferroptosis.

Glutathione (GSH) is a significant inhibitor and endogenous antioxidant involved in the process of ferroptosis. It primarily comprises glutamic acid, cysteine, and glycine [86]. Adequate levels of GSH can effectively counteract the heightened presence of reactive oxygen species within the body, thereby serving as an essential defense mechanism in safeguarding cells against various forms of oxidative stress (Figure 2a). Cystine-glutamate antiporter (System X_c_^−^) is a reverse transport system that acts as an antiporter and is made up of two subunits: the transporter subunit xCT (SLC7A11) and the regulatory subunit 4F2hc (SLC3A2). xCT translocates cystine into the cell, and 1:1 translocates glutamate out of the cell. Additionally, SLC3A2 is vital for maintaining the stability and subcellular location of SLC7A11 [87,88]. Upon entering the cytoplasm, cystine is quickly converted into cysteine, a significant building block for the production of GSH. Therefore, System X_c_^−^ is involved in the modulation of GSH synthesis so that GSH can protect cells from oxidative damage [89,90]. Furthermore, when GSH is present, glutathione peroxidase 4 (GPX4), a part of the glutathione peroxidase group, can act as a donor of electrons to transform the unstable lipid hydroperoxide (L-OOH) found in the cell membrane. This transformation converts the harmful malondialdehyde (MDA) derivative into a harmless lipid alcohol (L-OH), providing crucial protection for cells [91]. Researchers found that administering the ferroptosis inhibitor Liproxstatin-1 (Lip-1) reduced hyperalgesia and elevated iron levels and spinal lipid peroxidation in CCI rats, restoring imbalanced levels of GPX4 and acyl-CoA synthetase long-chain family member 4 (ACSL4) [78].

#### 4.1.3. Lipid Peroxidation Induces Ferroptosis

The peroxidation of PUFA-containing phospholipids in cell membranes is an essential step in ferroptosis [92]. Lipid peroxidation encompasses a multifaceted sequence of events involving the initiation of reactive oxygen species, the propagation of chain and chain-branched chain reactions, and the termination of free radical reactions. Free polyunsaturated fatty acids (PUFAs) could serve as crucial substrates for lipid metabolism in the physiological states [93,94]. These PUFAs undergo esterification into membrane phospholipids and oxidation to facilitate the transmission of ferroptosis signals [95] (Figure 2b). Meanwhile, lipid peroxidation is also involved in glutamine metabolism. A large number of oxidized and lipidated PUFAs are direct inducers of ferroptosis in cells. Phosa-phatidylethanolaminse (PE) with arachidonic acid (AA/AdA) is an essential phospholipid in cell membranes that triggers ferroptosis. ACSL4 facilitates the attachment of unbound AA/AdA to CoA to create AA-CoA/AdA-CoA derivatives when there is an abundance of ROS. Lysophosphatidylcholine acyltransferase 3 (LPCAT3) catalyzes the esterification of AA-CoA/AdA-CoA to AA-PE/AdA-PE in a subsequent step [96]. Ultimately, arachidonate 5-lipoxy-genase (ALOXs) enzymes convert AA-PE/AdA-PE into phospholipid hydroperoxides AA-OOH-PE/AdA-OOH-PE, which results in cell ferroptosis [97].

### 4.2. Ferroptosis and Neuroinflammation

Ferroptosis, especially iron homeostasis, is intimately associated with neuroinflammation [98]. Proteins involved in iron metabolism, including hepcidin, DMT1, TfR, and FPN1, are present in microglia, and their expression can be influenced by inflammatory stimuli (Table 1). The individual presence of LPS and β-amyloid (Aβ) would cause a great increase in DMT1 and ferritin in microglia with higher iron absorption and buildup in microglia [99]. Furthermore, the use of interferon-γ (IFN-γ) by itself would induce an upregulation in iron levels in microglia with a significant rise in the mRNA levels of TNF-α and iNOS [100]. Upon TNF-α stimulation, microglia show an elevation of DMT1 and inhibition of FPN1 expression, inducing higher iron uptake and retention within the cells [101].

The accumulation of iron in microglia, along with inflammatory agents, further exacerbates neuroinflammation. The existence of neuroinflammation in the pathogenesis of Sanfilippo syndrome (SP) has been suggested [102,103]. Coculturing heparan sulfate oligosaccharides from the urine of SP patients with microglia induces a downregulation of the expression of FPN1 in microglia, resulting in iron accumulation with the released inflammatory chemicals, finally triggering the robust neuroinflammatory response [104]. The accumulation of iron in microglia, along with inflammatory substances, exacerbates the advancement of neuroinflammation. Following the induction of a microglial iron overload by ferric ammonium citrate (FAC) and subsequent treatment with LPS, microglia are stimulated to produce increased levels of IL-1β and TNF-α [105]. Adding Fe^2+^ to LPS markedly enhances ROS generation in microglia compared to treating with LPS alone. The production of ROS in microglia is raised by Fe^2+^ in a non-phagocytic cell oxidase (NOX)-dependent manner. Exposure to Fe^2+^ and LPS can elevate the ROS in microglia, while the downregulation of NOX2 and NOX4 induces a reduction in the production of ROS [106]. Utilizing NOX2 inhibitors or NOX2 knockout mice results in a reduction in superoxide and ROS generation in microglia stimulated by Fe^2+^, ultimately decreasing neurotoxicity [107]. The accumulation of iron in microglia enables the activation of NOX, resulting in the development of neuroinflammation. Thus, inhibiting the accumulation of iron in microglia could attenuate the progression of neuroinflammation [108].

**Table 1:** Different agents targeting neuroinflammation in ferroptosis-related immune responses.

Targets	Effects on Ferroptosis	Effects on Neuroinflammation	References
LPS and Aβ	DMT1 and ferritin ↑	Pro-inflammatory cytokines ↑	[99]
IFN-γ	Microglia iron ↑	TNF-α and iNOS mRNA expression ↑	[100]
FAC and LPS	Microglia iron ↑	IL-1β and TNF-α ↑	[105]
NOX	Microglia iron ↑	Superoxide and ROS ↑	[106]
JNK pathway	DMT1 ↑, FPN1 ↓	IL-6 ↑	[109]

LPS, lipopolysaccharide; Aβ, β-amyloid; DMT1, divalent metal transporter 1; IFN-γ, interferon-γ; TNF-α, tumor necrosis factor-α; iNOS, inducible nitric oxide synthase; FAC, ferric ammonium citrate; IL-1β, interleukin-1β; NOX, non-phagocytic cell oxidase; ROS, reactive oxygen species; JNK, c-Jun N-terminal kinase; FPN1, ferroportin-1; IL-6, interleukin-6.

Microglia, an essential immune cell in the nervous system, is vital for activating the iron metabolism pathway by regulating the inflammatory response. This activation subsequently results in intracellular iron overload, inducing neuronal ferroptosis and the exacerbation of disease damage [110]. Furthermore, the disturbances in cellular iron metabolism that induce ferroptosis can also contribute to the polarization of microglial pro-inflammatory phenotypes, thereby intensifying the inflammatory response [111]. Nitrogen-doped graphene quantum dots (N-GQDs) have been found to have widespread applications in the areas of medical science and brain research. Experiments of in vitro models have shown that treating N-GQDs led to a decreased GSH, SLC7A11, and GPX4 in microglia but increased Fe^2+^, ROS, and lipid peroxide levels. These findings suggest that N-GQDs can induce ferroptosis in microglia. Moreover, N-GQDs have been discovered to increase the levels of pro-inflammatory factors IL-1β and TNF-α in microglia. As a result, the presence of pro-inflammatory substances may cause neuroinflammation to develop in microglia undergoing ferroptosis [112]. Therefore, the activation of pro-inflammatory molecules would result in neuroinflammation after ferroptosis in microglia. The accumulation of iron in microglia led to an upgrade of IL-6 mRNA expression. Stimulation of the JNK pathway by IL-6 leads to the upregulation of DMT1, while downregulating FPN1 finally results in increased iron buildup in microglia [109]. The accumulation of iron in microglia has caused an increase in TNF expression, resulting in the promotion of the M1 phenotype in microglia and the reduction in the M2 phenotype, ultimately hindering the recovery process following spinal cord injury [113].

Following the injection of LPS into the peritoneum of older mice, an increase in heme oxygenase-1 (HO-1) expression in microglial cells results in the buildup of iron. Moreover, increasing HO-1 in microglia could cause higher levels of ROS and inhibition of GPX4 [114]. Knockdown of the ACSL4 gene in microglia reduces their vulnerability to ferroptosis and decreases the production of pro-inflammatory cytokines [115]. RSL3 inhibits lipid peroxidation by inhibiting the enzymatic activity of GPX4, resulting in the induction of cell ferroptosis [87]. Microglia treated with RSL3 exhibit elevated mRNA levels of pro-inflammatory TNF-α, IL-6, and IL-1β, revealing a connection between the activation of microglia ferroptosis and an increase in inflammatory markers [116]. In contrast, resveratrol boosts SLC7A11 expression via Nrf2 signaling, leading to the inhibition of rotenone-induced microglia ferroptosis [117]. Therefore, blocking microglial lipid peroxidation and System Xc- has the potential to halt the advancement of neuroinflammation, which suggests that the occurrence of ferroptosis is related to changes in the inflammatory micro-environment of neuropathic pain.

### 4.3. Ferroptosis and Neuropathic Pain

How to attenuate neuropathic pain by inhibiting cellular ferroptosis has become a prominent topic in pathological research during both development and maintenance periods. Another stream of research has suggested that the enzyme GTP cyclohydrolase I (GCH1) may have a crucial role in activating microglia, which is essential in the onset of neuropathic pain [118]. The analysis of small RNA sequencing has shown 13 miRNAs with differential expressions in GCH1-KD cells. DE miRNAs primarily target genes involved in the PI3K-Akt signaling pathway, peroxisome function, and ferroptosis [118]. Ferroptosis induces neuropathic pain by decreasing the activity of neurons and astrocytes in the spinal dorsal horn [107]. Neuropathic pain induced by CCI is accompanied by the buildup of iron, elevated levels of lipid peroxidation, and disruption of ACSL4 and GPX4 [119]. Furthermore, aberrant morphological alterations in mitochondria, including shrinkage and membrane rupture, are confirmed by transmission electron microscopy [119].

Additionally, treating CCI rats with Lip-1 could reduce sensitivities, decrease iron levels, lower spinal lipid peroxidation, normalize GPX4 and ACSL4 levels, and prevent morphological changes in mitochondria caused by CCI [120]. Gallic acid exacerbates chronic pain and depression symptoms by inhibiting P2X7 receptor-induced ferroptosis in CCI rats [121]. Increased levels of Nox4 in DRG can trigger ferroptosis, resulting in neuropathic pain, while Methyl ferulic acid (MFA) can relieve neuropathic pain by blocking the upregulation of Nox4 in DRG [122]. EA inhibits ferroptosis through modulation of the SAT1/ALOX15 pathway for the management of neuropathic pain [123]. Different changes in biochemistry and physical characteristics are linked to ferroptosis in the spinal cord and DRG tissues of CFA rats [124]. The modifications consist of excess iron, increased lipid oxidation, disruptions in ACSL4 and GPX4 levels, and irregular changes in the structure of mitochondria [125]. Administering Lip-1 intrathecally reverses the ferroptosis-related alterations and relieves the mechanical and thermal hypersensitivities in CFA rats [125]. Enhanced levels of SIRT2 alleviate neuropathic pain triggered by SNI through increasing FPN1 expression, decreasing iron-induced lipid peroxidation, and maintaining the expression of GPX4 and ACSL4 to inhibit ferroptosis in the spinal dorsal horn of SNI rats [126].

### 4.4. Ferroptosis and α7nAChR

Stimulation of the α7nAChR receptor by electroacupuncture at the hindlimb Zusanli (ST36) acupoint inhibits ferroptosis elicited by LPS in alveolar epithelial cells, inducing a decrease in the pulmonary inflammatory response generated by LPS [127]. Hepcidin is a critical regulatory molecule that regulates iron ions in the body, and it is also an important regulatory protein of the essential transporter FPN for intracellular iron efflux. It degrades FPN via the specific modulation of FPN–hepcidin–FPN axis internalization, thereby modulating the iron homeostasis. Inflammatory response, hypoxia, insufficient red blood cell production, and hemolysis can all enhance hepcidin expression [128]. Iron absorption in microglia during neuroinflammation is regulated by hepcidin binding to FPN and the transcription levels of FPN or TfR under cholinergic impact [129]. An unconfirmed communication pathway links the α7nAChR signaling with the transferrin receptor–hepcidin–FPN pathway. One could suggest that this route goes both ways, with hepcidin–FPN signaling having a modulatory impact on the α7nAChR cascade [129]. Zhao et al. [130] reported that α7nAChR activation could enhance the ferroptosis pathway during ZIKA virus (ZIKV) infection. The administration of Hemin significantly increased the expression of HO-1, providing strong evidence for the crucial involvement of HO-1 in ferroptosis triggered by α7nAChR. Both intracellular Fe^2+^ and lipid peroxide levels were upregulated by α7nAChR activation, indicating an activated ferroptosis pathway. The activation of α7nAChR caused a decrease in the levels of Nrf2 and GPX4, which are two critical regulators of cellular resistance to mitigate lipid peroxidation and ferroptosis.

In conclusion, research on the relationship between α7nAChR and ferroptosis in neuropathic pain is still relatively insufficient. However, studies have shown that α7nAChR may hold tremendous potential in enhancing the necessarily required therapeutic effects for neuroinflammation and ferroptosis-mediated neuropathic pain. Further research of microglial α7nAChR will be conducive to the development of safe and efficient new drugs to relieve neuropathic pain.

## 5. Conclusions and Perspectives

**Figure 3:**
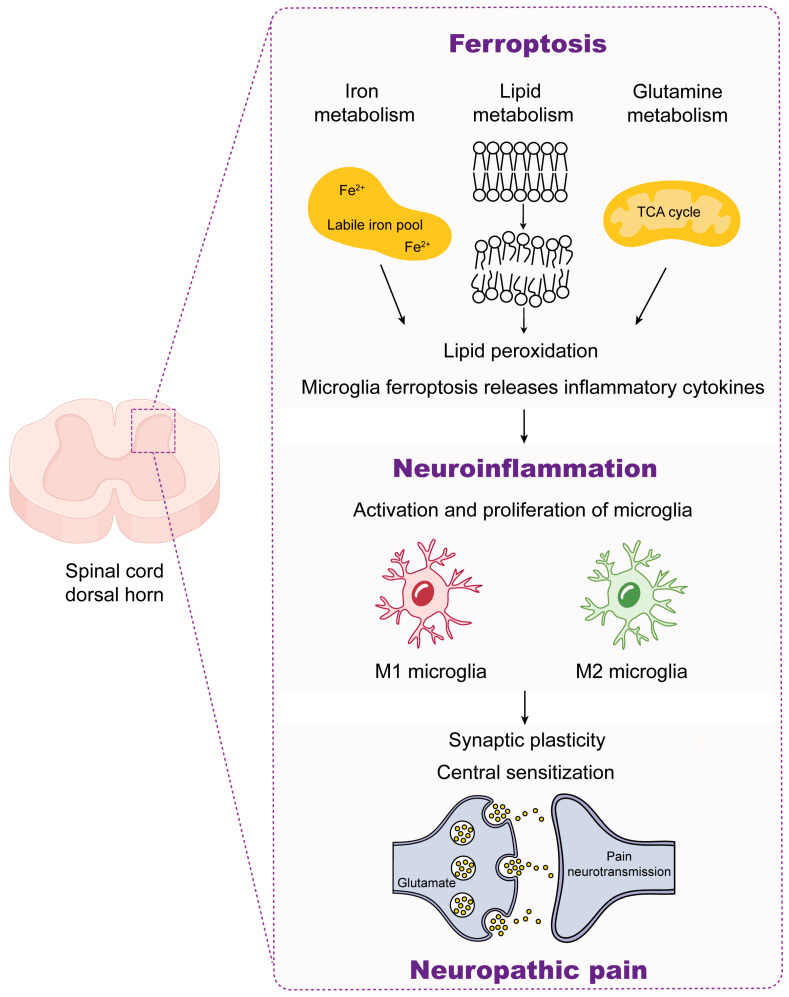
Schematic showing the mechanism of ferroptosis–neuroinflammation–neuropathic pain. Neuroinflammation can be caused by microglia iron accumulation, phospholipid peroxidation, or disturbed glutamine metabolism, releasing inflammatory cytokines. The activation and proliferation of microglia orchestrate neuroinflammation, which in turn can strongly modulate synaptic transmission and lead to central sensitization. Additionally, neuroinflammation plays an essential role in the development and progression of neuropathic pain.

The complicated pathogenesis of neuropathic pain significantly hinders the advancement of its treatment strategies. Recent research has indicated a close association between ferroptosis and neuropathic pain, but the underlying mechanisms are unclear and require further investigation. In this review, we discuss the key role of neuroinflammation in the development of neuropathic pain and summarize the relationship between α7nAChR, ferroptosis, and neuropathic pain. The interaction between ferroptosis in microglia and neuroinflammation is evident, as neuroinflammation can induce ferroptosis in microglia, and in turn, ferroptosis exacerbates the inflammatory response of microglia (Figure 3). Additionally, α7nAChR gating may serve as a shared target for both ferroptosis and neuroinflammation. Therefore, a new strategy for treating neuropathic pain may be to develop combined treatments that target α7nAChR activation, suppress microglia ferroptosis, and improve neuroinflammation.

In summary, the close association between neuropathic pain and ferroptosis necessitates further investigation into the underlying mechanisms. The interaction of neuroinflammation, aided by microglial α7nAChR, and its association with ferroptosis offer a promising treatment target for neuropathic pain. The specific complement molecules that ferroptosis mediated by α7nAChR have not been fully elucidated, but the future of this approach is very bright owing to technological advances and an increasing body of experimental evidence.

## Abbreviations

AA/AdA	Arachidonic acid
ACSL4	Acyl-CoA synthetase long-chain family member 4
ALOX	Arachidonate lipoxygenase
Aβ	β-amyloid
CAP	Cholinergic anti-inflammatory pathway
CCI	Chronic constriction injury
CFA	Complete Freund’s adjuvant
CNS	Central nervous system
DMT1	Divalent metal transporter 1
DRG	Dorsal root ganglion
EA	Electroacupuncture
FAC	Ferric ammonium citrate
FPN1	Ferroportin-1
GABAAR	γ-aminobutyric acid type A receptor
GlyR	Glycine receptor
GPX4	Glutathione peroxidase 4
GSH	Glutathione
IASP	International Association for the Study of Pain
IFN-γ	Interferon-γ
IL-10	Interleukin-10
IL-1β	Interleukin-1β
IL-6	Interleukin-6
iNOS	Inducible nitric oxide synthase
LIP	Labile intracellular iron pool
Lip-1	Liproxstatin-1
LPCAT3	Lysophosphatidylcholine acyltransferase 3
LPS	Lipopolysaccharide
MDA	Malondialdehyde
MFA	Methyl ferulic acid
nAChR	Nicotinic acetylcholine receptor
NF-κB	Nuclear factor-κB
NGF	Nerve growth factor
NOX	Non-phagocytic cell oxidase
Nrf2	Nuclear factor E2-related factor 2
PE	Phophatidyl ethanolamine
PG	Prostaglandin
PUFAs	Polyunsaturated fatty acids
RCD	Regulated cell death
ROS	Reactive oxygen species
RSL3	Ras-selective-lethal compound 3
SCP2-1	Polysaccharides of Schisandra Chinensis Fructus
SNI	Spared nerve injury
SP	Sanfilippo syndrome
STEAP3	Six-transmembrane epithelial antigen of the prostate 3
TBI	Transferrin-binding iron
TfR1	Transferrin receptor 1
TGF-β	Transforming growth factor-β
TNF-α	Tumor necrosis factor-α
5-HT3R	5-hydroxytryptamine 3 receptor
